# Observation Quality Assessment and Performance of GNSS Standalone Positioning with Code Pseudoranges of Dual-Frequency Android Smartphones †

**DOI:** 10.3390/s21062125

**Published:** 2021-03-18

**Authors:** Umberto Robustelli, Jacek Paziewski, Giovanni Pugliano

**Affiliations:** 1 Department of Engineering, Parthenope University of Naples, 80133 Naples, Italy; umberto.robustelli@uniparthenope.it; 2 Department of Geodesy, Faculty of Geoengineering, University of Warmia and Mazury in Olsztyn, Oczapowskiego 1, 10-719 Olsztyn, Poland; 3 Department of Civil, Architectural and Environmental Engineering, University of Naples Federico II, via Claudio 21, 80125 Naples, Italy; giovanni.pugliano@unina.it

**Keywords:** GNSS, smartphone, absolute positioning, GNSS noise assessment, Android, Galileo, GPS

## Abstract

The new generation of Android smartphones is equipped with GNSS chips capable of tracking multi-frequency and multi-constellation data. In this work, we evaluate the positioning performance and analyze the quality of observations collected by three recent smartphones, namely Xiaomi Mi 8, Xiaomi Mi 9, and Huawei P30 pro that take advantage of such chips. The analysis of the GNSS observation quality implies that the commonly employed elevation-dependent function is not optimal for smartphone GNSS observation weighting and suggests an application of the $C/N_{0}$-dependent one. Regarding smartphone code signals on L5 and E5a frequency bands, we found that they are characterized with noticeably lower noise as compared to E1 and L1 ones. The single point positioning results confirm an improvement in the performance when the weights are a function of the $C/N_{0}$-rather than those dependent on the satellite elevation and that a smartphone positioning with E5a code observations significantly outperforms that with E1 signals. The latter is expressed by a drop of the horizontal RMS from 8.44 m to 3.17 m for Galileo E1 and E5a solutions of Xiaomi Mi 9 P30, respectively. The best positioning accuracy of multi-GNSS single-frequency (L1/E1/B1/G1) solution was obtained by Huawei P30 with a horizontal RMS of 3.24 m. Xiaomi Mi 8 and Xiaomi Mi 9 show a horizontal RMS error of 4.14 m and 4.90 m, respectively.

## 1. Introduction

The ubiquity and high positioning performance of recent smartphones have widely expanded their primary application that was personal navigation [1]. As a consequence, the door to novel areas of market, industry, and science has been opened for smart, handheld, and low-cost GNSS devices [2,3,4]. As it was reported by European GNSS (Global Navigation Satellite System) Agency, smartphones are now dominating the installed base of devices that are equipped with GNSS chips [5]. Such progress induced the scientific community to draw special attention to GNSS observations derived from smartphones. As a result, a great deal of effort has been put into the development of observational and stochastic models that are suited to process smartphone GNSS observations [6,7]. These algorithms address the specific limitations of smartphone GNSS observations such as the low suppression to multipath and high observational noise highlighted by Riley et al. [8], the carrier phase discontinuity that is driven by duty-cycle analyzed by Paziewski et al. [9] and an existence of unwanted biases that destroy integer properties of phase ambiguities showed by Humphreys et al. [10] and Li and Geng [11].

Several researchers have already proved the feasibility of precise positioning with smartphone GNSS observations under these specific conditions. In one of the primary papers, Realini et al. [12] evaluated a short-baseline positioning without an integer ambiguity resolution and concluded that it is achievable to reach a decimeter-level accuracy in a relative mode with observations collected by a Nexus 9 smart device. Geng and Li [13] showed a centimeter-level accuracy of the relative positioning with an integer ambiguity; fixing this, however, required a priori calibration and correction of initial phase biases. Wen et al. [14] demonstrated Precise Point Positioning with smartphone and survey-grade GNSS antenna. Wanninger and Heßelbarth [15] proved that it is feasible to reach a coordinate precision of a few centimeters in a static relative positioning with GPS L1 observations collected by Huawei P30 and an embedded antenna. More recently, Paziewski et al. [16] presented a centimeter-level precision of the smartphone to smartphone positioning and Gao et al. [17] reported a subcentimeter accuracy of the short baseline RTK positioning with Xiaomi Mi 8 and Huawei P30 smartphones taking advantage of the linear fitting to restore the integer property of phase ambiguities.

Nevertheless, there is still a great need to evaluate an accuracy level that may be reached with current smart devices in a single point positioning (SPP) fed with code pseudoranges [18]. This is justified by the fact that even now there are only a few smartphones that offer continuous high quality phase observations. On the other hand, we may recognize a number of real-time or post-processing position-based applications which do not require a centimeter-level precision that may be reached only with phase observations [19,20]. Moreover, several manufacturers have recently released smart devices equipped with chipsets that acquire code pseudoranges not only on the first frequency but also on the second one, e.g., L5/E5a as in the case of GPS and Galileo system, respectively. As it was proved for geodetic receivers, code signals on L5 and E5a frequency bands are characterized with noticeably lower noise as compared to E1/L1 ones [21,22].

In a view of these conditions and expectations, this paper aims at the evaluation of the single point positioning performance of the most recent smartphones that track dual-frequency multi-constellations GNSS signals. We analyze potential progress in this term with respect to preliminary studies performed with early smartphones. We investigate the benefit from the integration of multi-constellation signals and analyze selected observation weighting schemes. We verify the hypothesis that smartphone positioning with L5/E5a code observations may significantly outperform that with L1/E1 signals.

This paper is organized as follows: in the next section, we describe the data collection and present the experiment design. In the third section, we analyze the GNSS observation quality in terms of signal power and observational noise. The positioning assessment is given in the fourth section, and this is followed by the discussion and conclusions that are given in the last section.

## 2. Data Collection and Experiment Design

We evaluate the positioning performance and analyze the quality of observations collected by three recent smartphones, namely Xiaomi Mi 8, Xiaomi Mi 9, and Huawei P30 pro. All devices track dual-frequency multi-constellation (GPS, Galileo, BDS, and GLONASS) signals. We note that Xiaomi Mi 9 only collects code pseudoranges, whereas Huawei P30, Xiaomi Mi 8 allow an acquisition of phase and code measurements. Since phase observations are not employed in a standard solution with a single point positioning mode, we neither evaluate their quality nor use them in the positioning assessment.

GNSS observations were collected on 11 December 2019 over a time span of 5 h (approximately 8–13 UTC) using Geo++ RINEX Logger ver. 2.1.6 application that runs on an Android system [23]. Since 3G and 4G signals may use the L-band and therefore can interfere with GNSS signals, SIM cards were not inserted in the devices during data collection.

**Figure 1:**
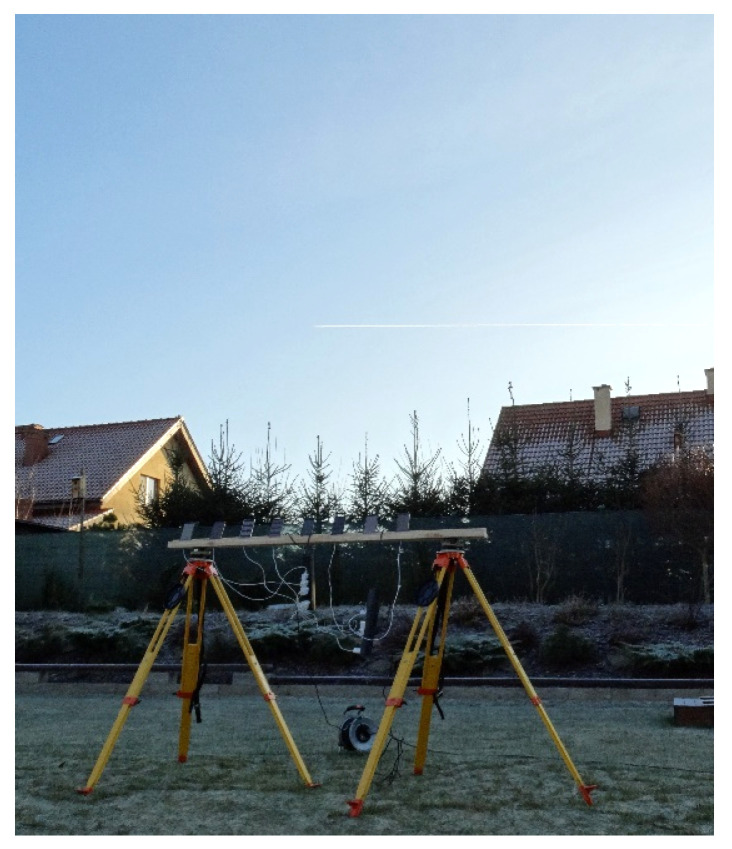
Smartphones during GNSS data collection.

The smartphones were centered over the temporal sites using tribrachs and tripods as showed in Figure 1. The devices were separated by a distance of 20 cm; therefore, we may consider the observational conditions as the same for each device. The ground truth coordinates of the temporal sites were determined in a static relative positioning using observations collected by the geodetic receivers and processed with multi-purpose GNSS processing software [24].

In Table 1, we show the average number of satellites that were tracked on consecutive frequency bands. A careful inspection of the table allows us to conclude that the most reasonable scenarios that can be used for the comparison of the smartphone positioning performance, are single-frequency (SF) GPS and SF multi-GNSS (GPS+Galileo+GLONASS+BDS). This is reasoned by the fact that other frequency bands offer a low number of acquired satellites. In addition, the classical dual-frequency ionosphere-free linear combination is unsuitable in this case due to few GPS satellites that provide signals on the L5 frequency band and a low number of acquired Galileo satellites on E5a band for Xiaomi Mi 8 and Huawei P30.

The benefit from the application of L5/E5a code pseudoranges may be reliably verified only by using Galileo observations of the Xiaomi Mi 9 smartphone. In this case, the satellite tracking performance was comparable for both frequency bands, namely E1 and E5a. However, by observing Table 1, we can notice that the average number of acquired GPS satellites on L5 is much lower than that on L1 for all smartphones. For the Galileo constellation, we see how the Xiaomi Mi 8 has a low number of satellites tracked both on the E1 and on the E5a. For the Huawei P30, a positioning with the E1 may be regarded as even impossible since an average number of satellites over the session reached only 1.2.

Despite the fact that Doresa and Milena—a pair of Galileo spacecrafts launched into highly eccentric orbital planes—have proved their applicability to standard [25] and precise GNSS positioning [26], the impact of these satellites may be considered as negligible, since only one of them was tracked for a few epochs by a single smartphone (Xiaomi Mi 8) during the experiment.

## 3. Signal Quality Assessment

The performance assessment of a standalone positioning with code pseudoranges is preceded by a quality evaluation of GNSS signals acquired by the employed smart devices. As previous studies showed, GNSS observations tracked by smartphones are subject to several unwanted effects and suffer from substantial observation noise and low suppression to multipath effect [9,15,27]. Such outcomes are said to be related to low strength of the GNSS observations acquired by smartphones. This section provides selected characteristics of GNSS observations and verifies, with regard to the most recent smartphones, the common assumption that smartphone GNSS signals are of poorer quality with respect to these collected by geodetic grade receivers. Our investigations are focused on the carrier-to-noise density ratio ($C/N_{0}$) that characterizes the GNSS signal power and noise of code pseudoranges [10]. We assess the quality of code observations since their noise directly propagates into the position estimates in single point positioning.

**Figure 2:**
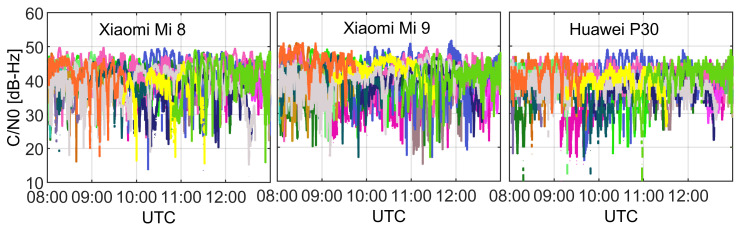
$C/N_{0}$ time series of GNSS signals collected by Xiaomi Mi 8, Xiaomi Mi 9, and Huawei P30 in left, middle, and right panels, respectively. The values corresponding to different satellites are distinguished by different colors.

We begin with the presentation of the time series of $C/N_{0}$ of GNSS signals that were tracked by analyzed smart devices in Figure 2. In the plots, the values corresponding to different satellites are distinguished by different colors. As we may read from the figure, the maximal power of acquired signals does not exceed 50 dB-Hz. We find that this value is significantly lower when compared to high grade receivers since the latter acquire signals with power reaching even over 50 dB-Hz [6,28,29]. What also transpires from the plots in Figure 2 is that the $C/N_{0}$ records are subject to frequent outliers characterized with ultra-low values. This effect holds true for all analyzed smartphones. We attribute this effect to higher susceptibility to multipath and worse elimination of outlying observations by smart devices with respect to high grade receivers and antennas.

**Figure 3:**
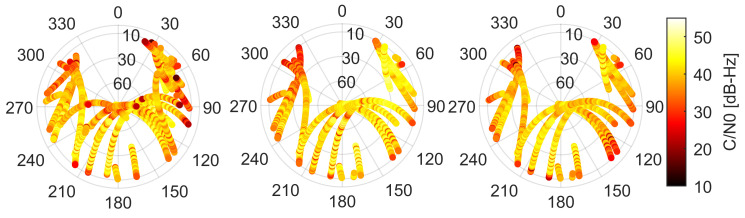
$C/N_{0}$ skyplots of GNSS signals collected by Xiaomi Mi 8, Xiaomi Mi 9, and Huawei P30 in left, middle, and right panels, respectively.

In the consecutive skyplots in Figure 3, we present the $C/N_{0}$ of GNSS observations as a function of azimuth and elevation of the satellite signal. The figure gives us the first impression of the $C/N_{0}$ dependence on signal elevation and verifies the assumption of azimuthal symmetry that holds true for high-grade receivers and antennas. Looking at the skyplots in Figure 3, we may conclude that this is not the case for GNSS observations collected by the smartphones. We do not just discover a low $C/N_{0}$ dependence on satellite elevation but also a clear azimuthal asymmetry of a signal gain. We also noticed a number of drops of $C/N_{0}$ that are unexpected for high elevations e.g., in the results for Xiaomi Mi 8 which are given in the left panel of Figure 3. Such results provide us with the first hint that, commonly employed, an elevation-dependent function may not be optimal for smartphone GNSS observation weighting and suggests an application to be the $C/N_{0}$-dependent one.

To complete the quality analysis of smartphone GNSS observations, now we focus on the stochastic properties of code pseudoranges. We take advantage of the satellite to receiver (absolute) observations that were double differenced in a time domain (DDTD). Such combination isolates the observation noise as the impact of low-frequency effects such as geometric distance, satellite orbits and clocks, receiver clock, atmospheric propagation errors as well as other time correlated effects are eliminated or significantly reduced [9,30]. Therefore, the variations of such combination residuals should be predominately attributed to the noise of observations.

**Figure 4:**
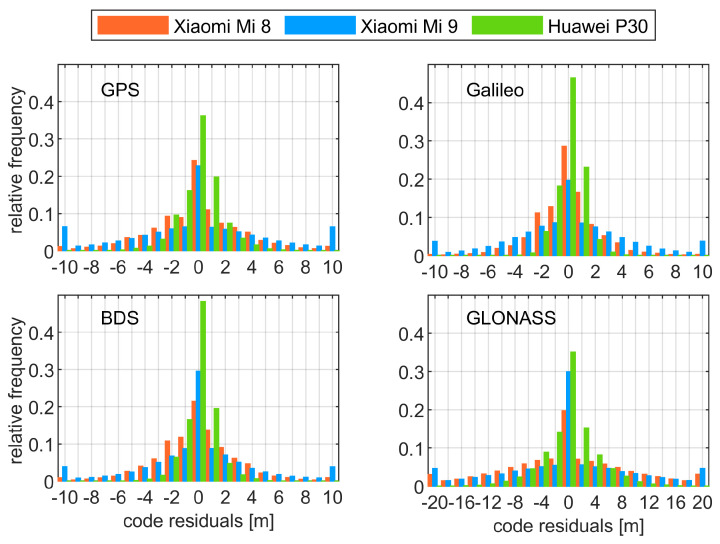
Histograms of the smartphone L1/E1/B1/G1 code residuals computed as the observations that were double differenced in a time domain. One should note that the GLONASS panel has different axis limits of code residuals when compared to the other panels. We also note an existence of outliers that are included in the outmost bins of code residuals.

In Table 2, we report the standard deviations (STD) of code pseudoranges obtained from the DDTD observations, whereas, in Figure 4, we show the distribution of DDTD observations for the consecutive smartphones and signals. We note that these statistics correspond to the undifferenced level, since they were normalized according to the error propagation law. We also recall that, for such observation combination, the multipath effect is greatly reduced.

Table 2 reveals significant divergences of observation noise among the devices, constellations, and frequency bands. As we may read, the employed smartphones are characterized with GPS L1 code noise fitting the range of 0.80 m–3.41 m. Slightly higher precision of observations was obtained for Galileo E1 and BDS B1 bands. On the other hand, STDs for GLONASS code signals were approximately twice of that and fit the range of 2.04–5.1 m, which is also clearly visualized in Figure 4. In Table 2, one can note a significant reduction of the code observation noise for GPS L5 and Galileo E5a frequency bands as compared to L1 and E1 ones, respectively. Such outcomes are coherent with those of high grade receivers, since it was already proved that L5 and E5a code signals exhibit a low observational noise [21]. If we confront the results of particular smartphones, we discover that Huawei P30 outperforms other devices in terms of the code pseudorange precision. Taking GPS L1 signal as an example, we report STD of 0.80 m for Huawei P30, over twice that for Xiaomi Mi 8, and fourfold for Xiaomi Mi 9. Such findings also hold true for other signals and constellations, since Xiaomi Mi 9 always gives the worst, and Huawei P30 always has the best performance in terms of the code noise.

**Figure 5:**
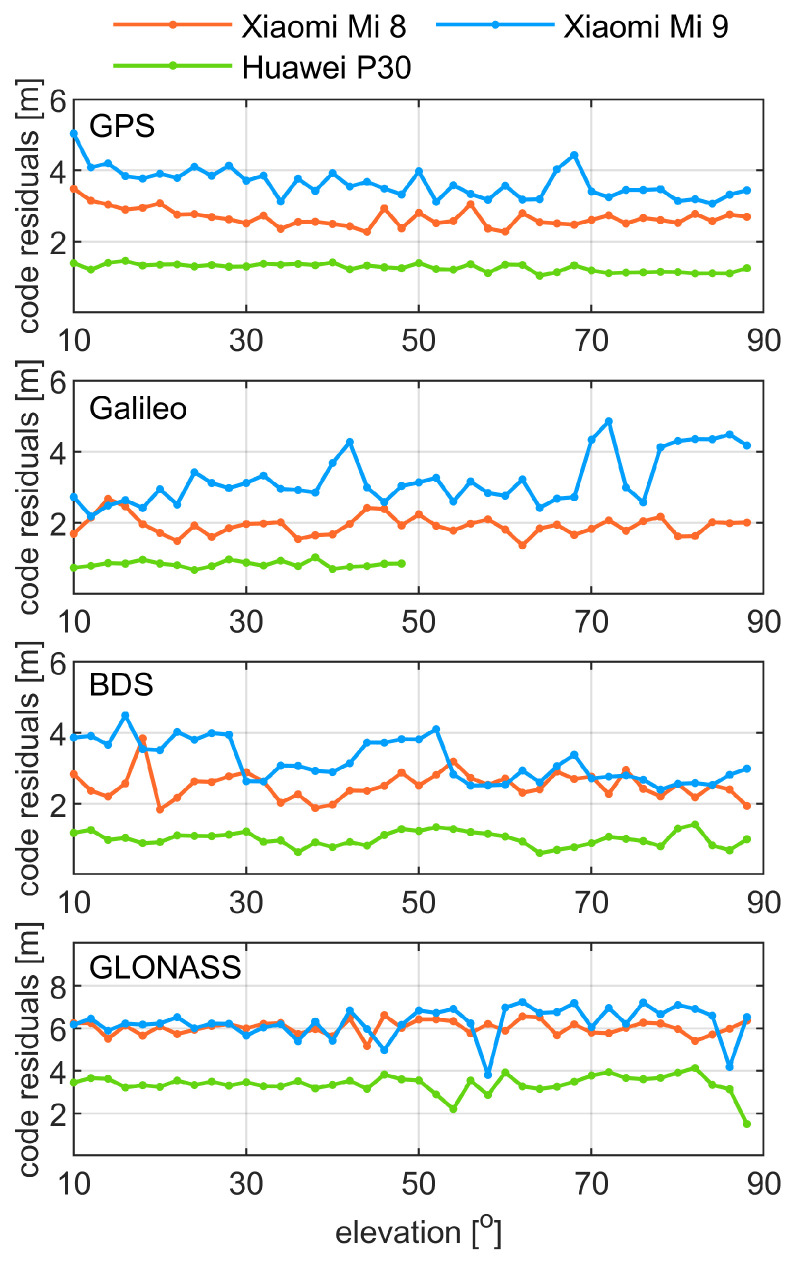
Elevation-dependent quality of code observations on GPS L1, Galileo E1, BDS B1, and GLONASS G1 frequency bands, respectively. Code residuals were computed as the observations that were double differenced in a time domain. The values are averaged over all satellites in 1${}^{\circ }$ bins. One should note that the GLONASS panel has different axis limits of code residuals when compared to the other panels.

To investigate the dependence of the code noise on satellite elevation, we present in Figure 5 the code residuals as a function of the satellite elevation. We recall that these residuals correspond to the observations that were double differenced in time domain. We also note that a 10${}^{\circ }$ elevation cut-off angle was employed, and the values were averaged in 1${}^{\circ }$ bins for the clarity of a presentation. In general, the plots show a low elevation dependence of the code noise. For all constellations, Huawei P30 and Xiaomi Mi 8 demonstrate almost an equal level of the code noise over the employed elevation range (10–90${}^{\circ }$). The results for Xiaomi Mi 9 exhibit a higher variability; however, we still cannot attribute this effect to the changing elevation angle of the satellite signals. Such findings hold true for all constellations, since in this term we did not notice any significant divergences between GNSS systems.

**Figure 6:**
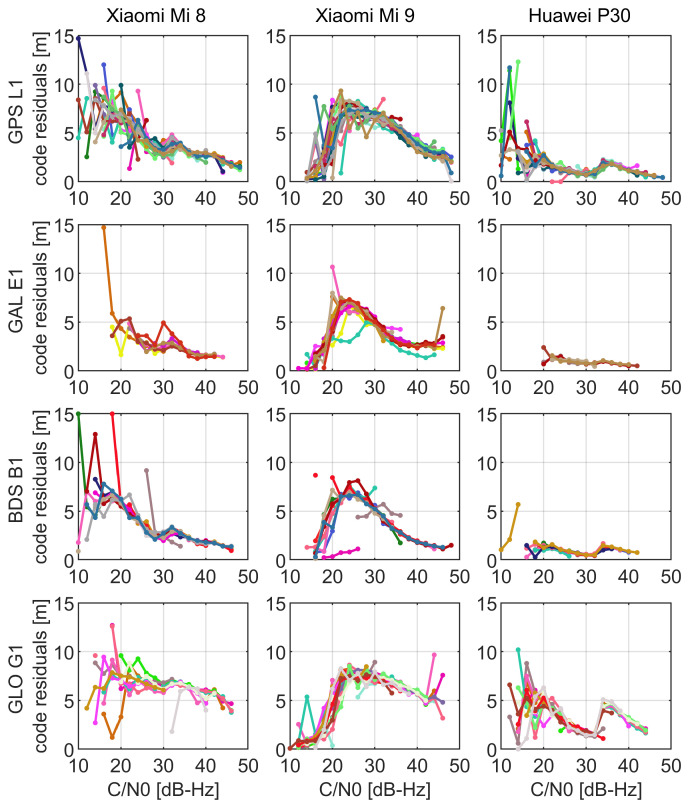
$C/N_{0}$-dependent quality of code observations on GPS L1, Galileo E1, BDS B1, and GLONASS G1 frequency bands in 1st, 2nd, 3rd, and 4th rows, respectively. The results corresponding to different smartphones are distinguished by columns. Different colors represent different satellites. Code residuals were computed as the observations that were double differenced in a time domain. The values are averaged for particular satellites in 1 dB-Hz bins.

In Figure 6, we show the dependence of a code noise on a signal carrier-to-noise density ratio. We distinguish the results between constellations and devices in consecutive panels and between the satellites by different colors. What one can read from the figure is that, despite the fact that $C/N_{0}$ presented in Figure 6 were averaged in 1${}^{\circ }$ bins, the values may still exhibit relative noise nature (e.g., code on GPS L1 of Xiaomi Mi 8 for $C/N_{0}$ in the range of 10–25 dB-Hz). However, if we carefully inspect Figure 6, we note that the smartphone code noise demonstrates a clear dependency on a signal strength. Recalling the results of the elevation-dependent quality of code observations that were given in Figure 5, we may conclude that the $C/N_{0}$ weighting scheme should be considered as superior to the elevation-dependent one. However, this will be validated in the positioning experiment in the next section. We also discover that the $C/N_{0}$ series of Xiaomi Mi 9 are of a different nature when compared to those of Xiaomi Mi 8 and Huawei P30. For the latter devices, we noticed that generally the highest noise corresponds to the signals with the lowest strength (up to 20 dB-Hz). Some small local peaks may be also detected as in the case of GLONASS signals of Huawei P30 around 35 dB-Hz. However, when it comes to the Xiaomi Mi 9 smartphone, we see that the noisiest observations are those with $C/N_{0}$ fitting the range of 20–30 dB-Hz. This is another finding, after the highest signal noise that proves a dissimilar performance of Xiaomi Mi 9 with respect to the other analyzed smartphones.

## 4. Performance Assessment of Single Point Positioning with Smartphone Code Pseudoranges

This section is devoted to the performance assessment of the positioning based on smartphone code observations. We analyze an accuracy level that may be reached with employed smartphones; we evaluate a potential benefit from the $C/N_{0}$-dependent weighting scheme and from the application of the signals that are transmitted on the second frequency band (L5/E5).

### 4.1. Positioning Methodology

The positioning was performed using the well-known multi-GNSS single point positioning algorithm fed with code pseudoranges [31,32,33]. When multi-constellation signals are integrated in the observational model, one should properly handle different system time scales. This may be performed by adopting GPS time scale as a reference time and modeling inter system time difference parameters as constant biases [34]. As a result, the observational equations for single-frequency multi-constellation point positioning, here generalized to frequency *n* of undifferenced observations between satellite and receiver, read as follows:

(1) $$P_{r,n}^{s,G}-\rho_{r,0}^{s,G}+c\cdot dt^{s,G}+b_{n}^{s,G}-d_{r,trop}^{s,G}-d_{r,n,ion}^{s,G}=e_{r}^{s,G}\cdot \delta X_{r}+c\cdot d{\bar{t}}_{r}+\epsilon_{r,n}^{s,G}$$

(2) $$P_{r,n}^{s,R}-\rho_{r,0}^{s,R}+c\cdot dt^{s,R}+b_{n}^{s,R}-d_{r,trop}^{s,R}-d_{r,n,ion}^{s,R}=e_{r}^{s,R}\cdot \delta X_{r}+c\cdot \left(d{\bar{t}}_{r}+dt_{r}^{R-G}\right)+\epsilon_{r,n}^{s,R}$$

(3) $$P_{r,n}^{s,E}-\rho_{r,0}^{s,E}+c\cdot dt^{s,E}+b_{n}^{s,E}-d_{r,trop}^{s,E}-d_{r,n,ion}^{s,E}=e_{r}^{s,E}\cdot \delta X_{r}+c\cdot \left(d{\bar{t}}_{r}+dt_{r}^{E-G}\right)+\epsilon_{r,n}^{s,E}$$

(4) $$P_{r,n}^{s,C}-\rho_{r,0}^{s,C}+c\cdot dt^{s,C}+b_{n}^{s,C}-d_{r,trop}^{s,C}-d_{r,n,ion}^{s,C}=e_{r}^{s,C}\cdot \delta X_{r}+c\cdot \left(d{\bar{t}}_{r}+dt_{r}^{C-G}\right)+\epsilon_{r,n}^{s,C}$$

where the superscript *s* denotes a particular satellite of the selected constellation, namely GPS (G), GLONASS (R), Galileo (E) or BDS (C); the subscript *r* states for the receiver; *P* is the code pseudorange in meters; $\rho_{0}$ is the geometric range between the satellite and a priori position of the station in meters; $dt^{sys}$ is the satellite clock correction for a particular satellite of a selected constellation in seconds (sys=G,R,E, or *C*); *b* denotes the signal specific satellite code bias in meters; $d_{trop}$ states for the tropospheric delay in meters; $d_{ion}$ denotes the ionospheric delay in meters; *e* is the line-of-sight vector from the user to the satellite; $d\bar{t}$ refers to the receiver clock correction combined with the receiver hardware delay in seconds; *c* is the speed of light in meters per second; $dt^{R-G},dt^{E-G},dt^{C-G}$ are GPS-GLONASS, GPS-Galileo, and GPS-BDS receiver inter system clock biases in seconds, respectively; δX refers to the vector of the corrections to a priori geocentric coordinates of the station $\left\{\delta x_{r},\delta y_{r},\delta z_{r}\right\}$ in meters, and ϵ corresponds to the combination of the non-modeled terms, specifically the multipath effect and the observational noise in meters. Finally, the vector of estimates reads as follows:

(5) $$x=[\delta x_{r},\delta y_{r},\delta z_{r},d{\bar{t}}_{r},dt_{r}^{R-G},dt_{r}^{E-G},dt_{r}^{C-G}]$$

Ionosphere delays are corrected by the broadcast ionospheric model [35], troposphere delays are modeled by the Saastamoinen model and GMF mapping function [36,37]. We employed orbits and clocks provided by Wuhan University [38] to show the performance level that may be reached when one employs precise products. The products provided by Center for Orbit Determination in Europe [39] were used to handle satellite code biases and to correct precise satellite clock offsets for single-frequency observations. The computations were performed with 5 s sampling interval and a cut-off angle of 10 degrees using the whole 5-h long time span of data collection that was described in Section 2. The parameters are estimated by least squares adjustment adopting a selected weighting scheme, namely elevation- or $C/N_{0}$-dependent one as described in [9].

### 4.2. Positioning Performance Comparison of the Smartphones and Benefits from the $C/N_{0}$-Dependent Weighting Scheme and Multi-GNSS Signals

The analysis carried out in the previous section implies that the commonly employed elevation-dependent function may not be advantageous for smartphone GNSS observation weighting and suggests an application of the $C/N_{0}$-dependent ones. Therefore, we begin with the evaluation of the advantage of the $C/N_{0}$ weighting function over the elevation-dependent one in the coordinate domain.

**Figure 7:**
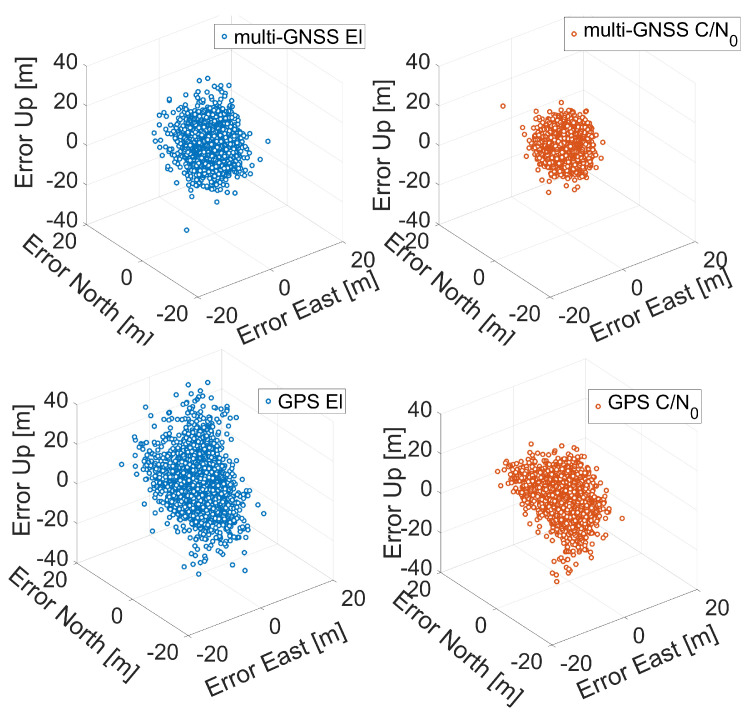
Three-dimensional coordinate error scatter plots for single point positioning of Xiaomi Mi 8. The first row corresponds to multi-GNSS solution while the second row to the GPS one. Blue markers represent errors when the solution was obtained using elevation-dependent weights; red circles represent errors obtained using $C/N_{0}$ weights.

**Figure 8:**
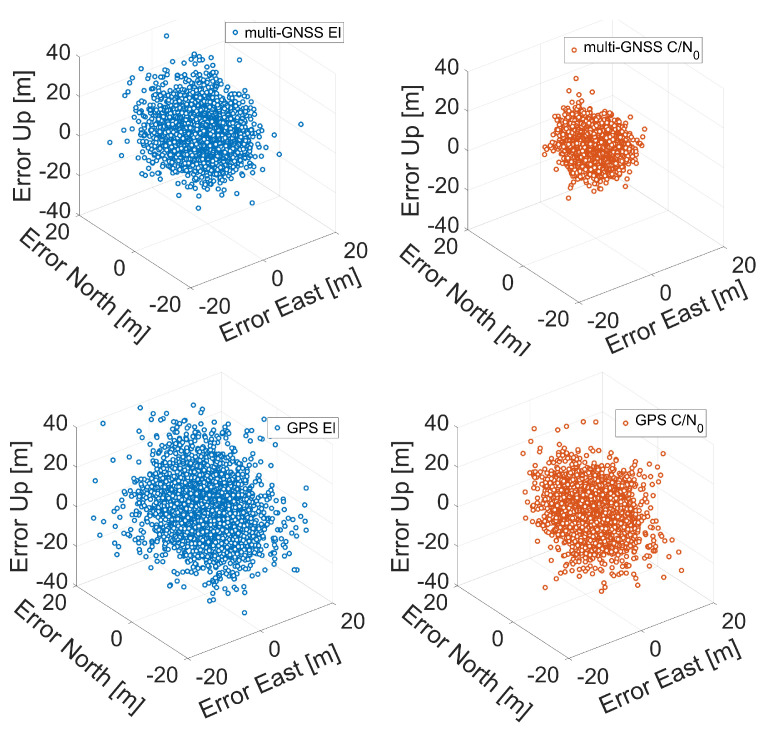
Three-dimensional coordinate error scatter plots for single point positioning of Xiaomi Mi 9. The first row corresponds to multi-GNSS solution while the second row to the GPS one. Blue markers represent errors when the solution was obtained using elevation-dependent weights; red circles represent errors obtained using $C/N_{0}$ weights.

**Figure 9:**
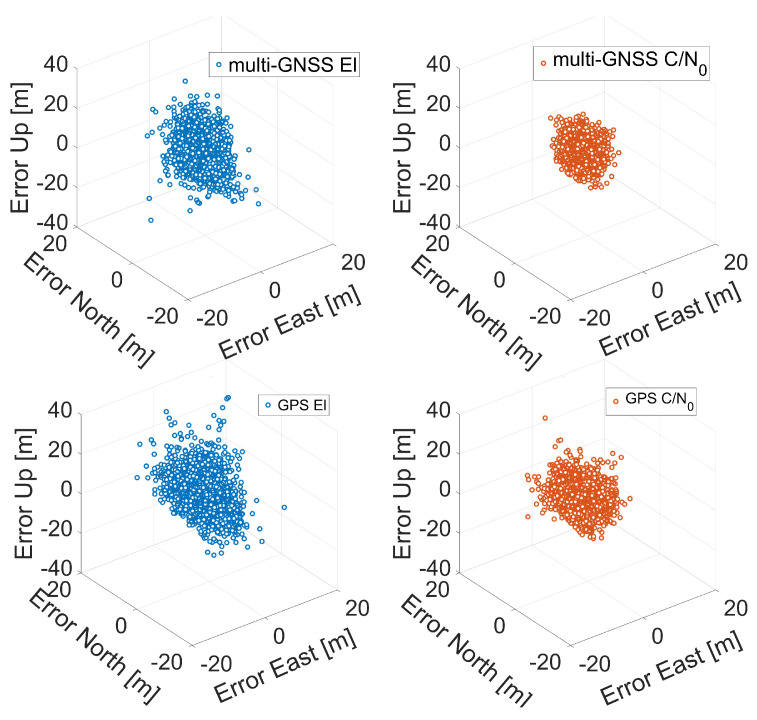
Three-dimensional coordinate error scatter plots for single point positioning of Huawei P30. The first row corresponds to the multi-GNSS solution while the second row to the GPS one. Blue markers represent errors when the solution was obtained using elevation-dependent weights; red circles represent errors obtained using $C/N_{0}$ weights.

In Figure 7, Figure 8 and Figure 9, the three-dimensional (3D) positioning error scatter plots for Xiaomi Mi 8, Xiaomi Mi 9, and Huawei P30 smartphones are shown, respectively. The first row of each figure shows the errors obtained using a multi-constellation solution fed with single-frequency observations (L1/E1/B1/G1), while the second row shows the errors obtained using GPS L1 C/A code signals. Blue markers represent errors when the solution was obtained using elevation-dependent weights while red circles represent error obtained using $C/N_{0}$-related weights. The figures clearly confirm that the application of $C/N_{0}$-dependent weighting function gives better results than those obtained with the elevation-dependent function: the red point cloud is always contained within the blue one.

**Figure 10:**
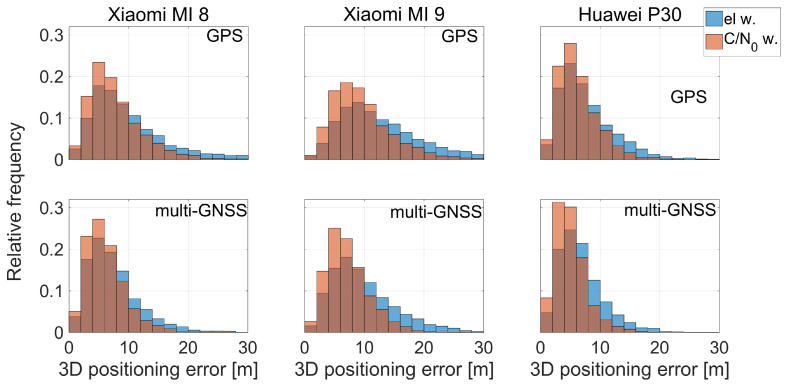
Three-dimensional coordinate error histogram for GPS and multi-GNSS solutions. The results achieved with the elevation-dependent weighting scheme are represented in blue while those obtained with $C/N_{0}$ weighting are represented in red. Each bin is 2 m wide. Xiaomi Mi 8, Xiaomi Mi 9, and Huawei P30 smartphones are shown in the left, middle and right columns, respectively. A cut-off threshold of 30 m is used to achieve a better visibility of higher probability region.

Figure 10 shows the 3D positioning error histogram for both GPS and multi-constellation solutions. The results achieved by using elevation-dependent weighting are represented in blue while in red those obtained with $C/N_{0}$-dependent weighting. Each bin is 2 m wide. Xiaomi Mi 8, Xiaomi Mi 9, and Huawei P30 smartphones are shown in the left, middle and right column, respectively. This figure confirms that the accuracy obtained using the $C/N_{0}$ weighting is higher than that obtained using the elevation one; the blue curves are always wider than the red ones; thus, the errors for the $C/N_{0}$-dependent scheme have a standard deviation lower than those obtained with the elevation-dependent scheme.

**Figure 11:**
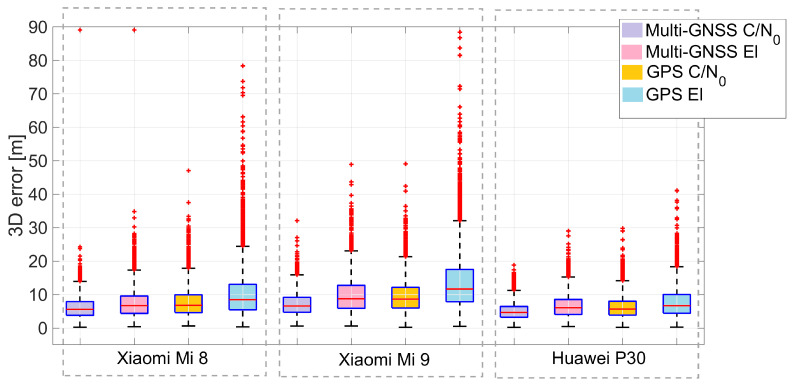
Three-dimensional coordinate error box-plots for GPS and multi-GNSS solutions with both $C/N_{0}$ and elevation-dependent weighting schemes. Xiaomi Mi 8, Xiaomi Mi 9, and Huawei P30 smartphones are shown in the first, second, and third panels, respectively. A cut-off threshold of 90 m is used to achieve a better visibility. Red crosses represent the outliers.

Figure 11 represent three-dimensional coordinate error box-plots for GPS and multi-GNSS SPP solutions with both $C/N_{0}$ and elevation weighting. Xiaomi Mi 8, Xiaomi Mi 9, and Huawei P30 smartphones are shown in the first, second, and third panels, respectively. Some errors for the Xiaomi Mi 9 are very high (occasionally greater than 150 m). To ensure a better view of the figure, a threshold of 90 m has been adopted. The light purple boxes represent the errors obtained with the multi-GNSS and $C/N_{0}$ weighting scheme, the pink boxes represent errors for multi-GNSS with an elevation weighting scheme. Orange and light blue boxes represent errors for the GPS approach with $C/N_{0}$ and elevations weighting functions, respectively. From the comparison of the boxes in the figure, the improvement provided by the use of weights as a function of $C/N_{0}$ is evident: the purple and orange boxes (relating to this type of observables weighing scheme) are more “compact” than the pink and light blue ones (related to weighing in function of satellite elevation) and positioned lower. This type of representation also allows us to highlight the best performance provided by the Huawei P30 compared to other smartphones even in relation to outliers, represented by the red cross in the figure.

Table 3 reports mean and standard deviations of 3D coordinate error for each smartphone for both weighting schemes. For all three smartphones, the results obtained with $C/N_{0}$-dependent strategy are always better than those obtained with elevation dependent ones. The best improvement is obtained for the Xiaomi Mi 9: with GPS solution, the mean error and STD are of 14.85 m and 17.15 m when elevation weighting is used with respect to 9.62 m and 5.15 m when $C/N_{0}$-dependent weighting is used. A good improvement is also achieved with the Xiaomi Mi 8: the mean error is reduced by about 3 m and STD is halved when only GPS satellites are used. Finally, the improvement is about 2 m when the Huawei P30 and the multi-GNSS strategy are considered.

**Figure 12:**
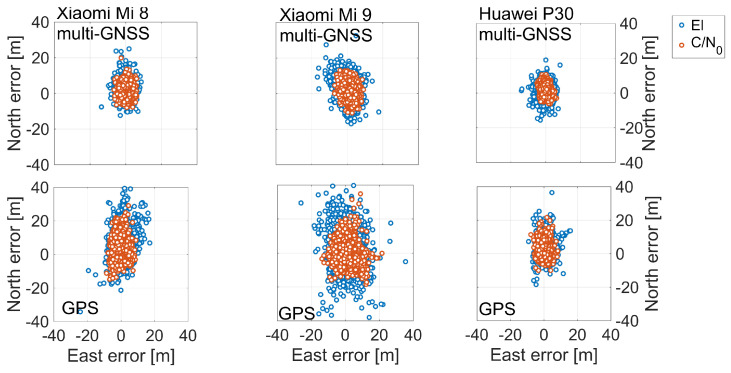
Horizontal coordinate component error scatter plots for single point positioning: elevation (represented in blue marker) versus $C/N_{0}$ (represented in red marker) dependent weighting schemes for multi-GNSS (first row) and GPS (second row) solutions. Xiaomi Mi 8, Xiaomi Mi 9, and Huawei P30 smartphones are shown in the left, middle, and right panels, respectively.

**Figure 13:**
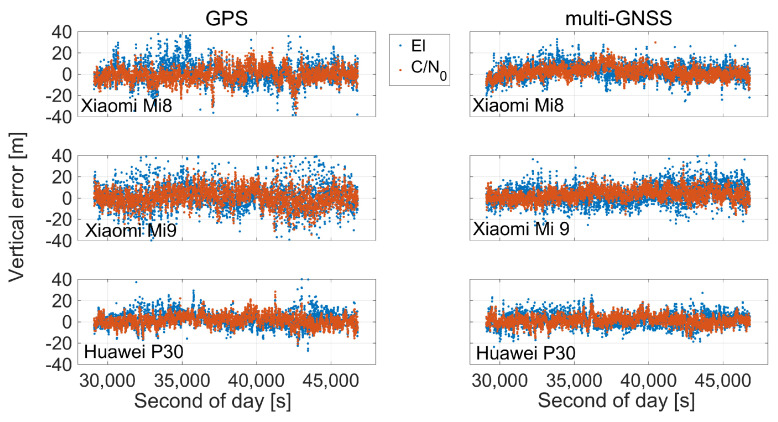
Time series of vertical coordinate component error for single point positioning: elevation (represented in blue marker) versus $C/N_{0}$ (represented in red marker) dependent weighting schemes for GPS (first column) and multi-GNSS (second column) solutions. Xiaomi Mi 8, Xiaomi Mi 9 and Huawei P30 smartphones are shown in the top, middle, and bottom rows, respectively.

This improvement in position accuracy is also evident by separating the horizontal component of the error from the vertical one as showed in Figure 12 and Figure 13. In Figure 12, the blue markers represent the errors obtained when the elevation weighting scheme is used, while the red markers represent the errors obtained when the $C/N_{0}$ weighting function is used. In the figure, multi-GNSS and GPS solutions are given in the first and the second rows, respectively, while Xiaomi Mi 8, Xiaomi Mi 9, and Huawei P30 smartphones results are distinguished by columns. Figure 13 shows the time series of vertical coordinate component error with the same representation of the previous figures. The red clouds showed in Figure 12 are all contained within the blue ones. This highlights how the horizontal component accuracy is improved when $C/N_{0}$ weighting is used. The figure also clearly shows the improvement that is achieved by using multi-constellation signals instead of GPS only that was, however, expected. What happens for the horizontal errors is exactly repeated also for the vertical component shown in Figure 13.

The next comparison we present is the one between the various smartphones. By observing the histograms shown in Figure 10, it can be immediately noted that the worst results in terms of three-dimensional error are obtained by the Xiaomi Mi 9: each histogram related to it has a wider tail. Vice versa, the Huawei P30 smartphone shows the best results: its histograms are narrower than the others. Figure 7, Figure 8, Figure 9, Figure 10, Figure 11, Figure 12 and Figure 13 show that the best positioning performance of Huawei P30 is in line with what is shown in the section related to the analysis of the observables since code pseudoranges of this smartphone exhibit the lowest noise.

The complete computation of all statistics is shown in Table 3 (where the mean and STDs of the positioning errors are listed) and Table 4 where the RMSs of the horizontal, vertical, and three-dimensional coordinate errors are reported. The results shown in the tables confirm what was previously stated. Here, for the sake of brevity, we will only comment on the results obtained using the best weighting scheme ($C/N_{0}$). The best performance is obtained by the Huawei P30. If the multi-GNSS signals are used, the horizontal, vertical, and three-dimensional RMS error are 3.24 m, 4.73 m, and 5.73 m, respectively. Even in the case of using only the GPS constellation, this smartphone shows the best performance with horizontal, vertical, and three-dimensional RMS errors of 4.47 m, 5.52 m, and 7.10 m, correspondingly. The performance of the Xiaomi Mi 8 is slightly worse than that of the Huawei P30 but still comparable: the errors of the Mi 8 are higher than those of the P30 by about 1.5 m (the maximum difference recorded is less than 2 m while the minimum is 0.9 m). Finally, the worst performances are obtained by the Xiaomi Mi 9 as predicted in the section where we analyzed the observables. For this smartphone, we get a three-dimensional RMS error of 10.92 m in the GPS configuration and of 8.05 m in the multi-GNSS configuration compared to the 7.10 m and 5.73 m obtained by the Huawei P30.

The results, as it was legitimate to expect, also highlight the clear improvement obtained when using the multi-constellation observations. The advancement is significant for all three smartphones: if we refer to the horizontal RMS error, we pass from the values equal to 5.61 m, 6.82 m, 4.47 m (for the Xiaomi Mi 8, Xiaomi Mi 9, Huawei P30, respectively) to the values equal to 4.14 m, 4.90 m, and 3.24 m (for the same smartphones). This type of improvement is also obtained for the vertical component.

### 4.3. Galileo Standalone Positioning: E5a versus the E1-Based Solution

The next analysis aims to verify if the positioning with the E5a code observations outperforms that with E1 ones as the former signals are of lower noise than the latter (cf. Table 2). To this end, we compare between the single point positioning results obtained using Galileo signals on the E1 and E5a frequency bands for the Xiaomi Mi 9 smartphone.

**Figure 14:**
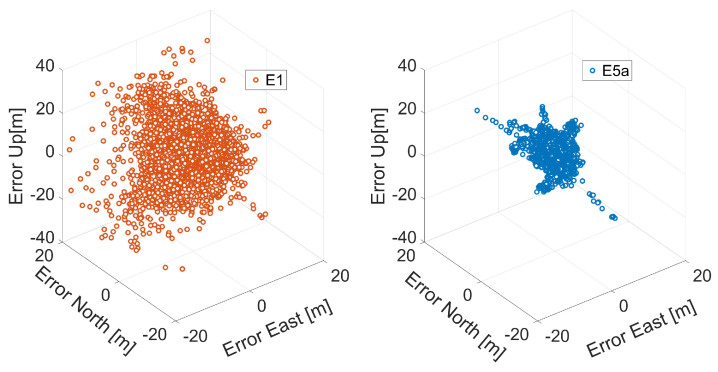
Three-dimensional coordinate error scatter plots for Galileo single point positioning of Xiaomi Mi 9. The left panel presents an E1-based solution, and the right panel corresponds to an E5a-based solution.

**Figure 15:**
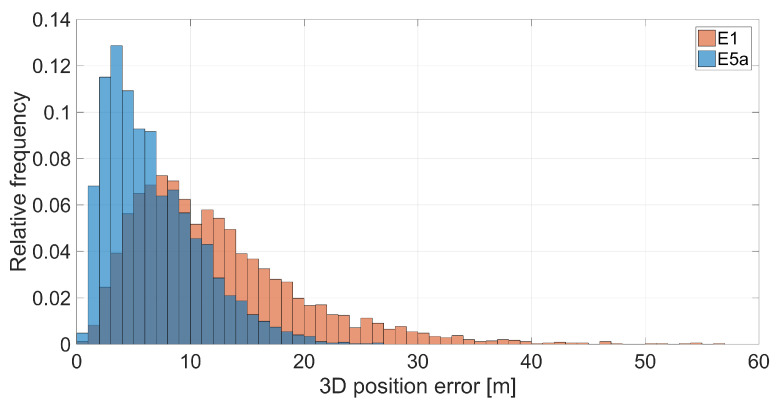
Three-dimensional coordinate error histogram for Galileo single point positioning of Xiaomi Mi 9. The results achieved with with E1 code observations are represented in red while those obtained with E5a ones are represented in blue. Each bin is 1 m wide.

In Figure 14, the three-dimensional positioning error comparison between E1 (left panel, red color) and E5a signals (right panel, blue color) of Xiaomi Mi 9 is shown. Figure 15 shows the corresponding three-dimensional position error histogram in the same color scheme. Each bin is 1 m wide. Both figures immediately point out how the accuracy obtained using the signals on the E5a frequency is higher than that obtained using the signals on E1. The blue curve (E5a error) is narrower than the red one (E1) in the histograms given in Figure 15. This conclusion is confirmed by the statistics given in Table 5. The table reveals that 3D-RMS is 14.34 m for Galileo single point positioning with E1 signals with respect to 7.91 m in the case of an E5a-based solution: the use of pseudoranges on E5a instead of the E1 frequency band led to a halving of the three-dimensional error.

**Figure 16:**
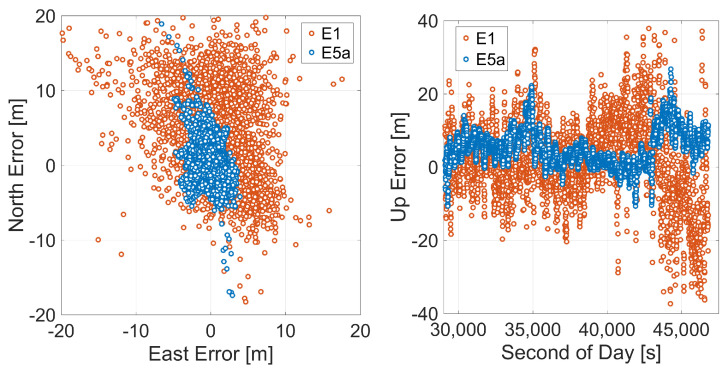
Horizontal and vertical coordinate component error scatter plots for Galileo single point positioning of Xiaomi Mi 9. Red markers represent an E1-based solution, while blue ones correspond to the E5a-based solution.

The improvement in position accuracy obtained using the E5a instead of the E1 code observations is also evident by separating the horizontal component of the error from the vertical one as showed in Figure 16. The horizontal error scatter plot and the vertical error time series are shown in the left and the right panels, respectively. The horizontal accuracy expressed in terms of root mean square of the horizontal error is of 8.44 m and 3.17 m for E1 and E5a-based SPP solutions, respectively (Table 5). With regard to vertical accuracy, the results confirmed the best performance of SPP with E5a observations with a RMS of the vertical component of 7.24 m compared to the 11.60 m of E1-SPP. The results are a confirmation of what is highlighted in the observation assessment section where we showed that, for the analyzed smartphones, the noise on the E5a is lower than that on the E1. These results are also coherent with the findings of Robustelli et al. [40], who attributed the better performance the E5a-based positioning as compared to the E1-based one, to the reduction of the multipath error.

## 5. Discussion

In this work, we assessed the quality of the observations collected by three selected recent smartphones, namely the Xiaomi Mi 8, Xiaomi Mi 9, and Huawei P30 pro and their single point positioning performance. First, we analyzed the $C/N_{0}$ of GNSS signals collected by the smartphones as a function of azimuth and elevation of satellites. We found both a low $C/N_{0}$ dependence on satellite elevation and a clear azimuthal asymmetry of signal gain. The second analysis carried out was focused on the stochastic properties of code pseudoranges. This analysis reveals significant divergences of observation noise among devices, constellations, and frequency bands. If we compare the results between the smartphones, we discover that Huawei P30 outperforms other devices in terms of code pseudorange precision while Xiaomi Mi 9 always gives the worst results. Regarding the different frequencies, we observed a significant reduction of code noise for GPS L5 and Galileo E5a frequency bands as compared to L1 and E1 ones. In fact, the standard deviation is reduced by a factor of approximately 4 for all three smartphones when using the L5 signal instead of L1 (0.51 m, 0.96 m and 0.19 m instead of 1.86 m, 3.41 m, and 0.80 m for Xiaomi Mi 8, Xiaomi Mi 9, and Huawei P30, respectively). A clear improvement is also obtained by using the E5a signal instead of E1 passing from 1.24 m, 2.77 m and 0.48 m to 0.54 m, 0.90 m, 0.21 m for for Xiaomi Mi 8, Xiaomi Mi 9 and Huawei P30, respectively.

Later, we investigated the dependence of the code noise on satellite elevation and on signal carrier-to-noise density ratio. We found a low elevation dependence of the code noise, while its dependency on the signal strength is clear. For Xiaomi Mi 8 and Huawei P30, we found that the highest noise corresponds to the signals with the lowest strength as expected, while the Xiaomi Mi 9 has the noisiest observations with $C/N_{0}$ in the range of 20–30 dB-Hz. The code noise analyses suggest that the $C/N_{0}$ weighting scheme should be considered as superior to the elevation-dependent one, and the expected performance of Xiaomi Mi 9 in terms of positional accuracy should be the worst.

Regarding positioning domain, several techniques were used: L1 GPS, E1 Galileo, L1 GLONASS single point positioning, L1 multi-constellation single point positioning, L5 GPS, and E5a Galileo single point both with elevation- and $C/N_{0}$-dependent weighting function. Based on the number of visible satellites shown in Table 1, we discuss here only the results for the most significant configurations, i.e., multi-constellation and GPS only L1 and Galileo E1 and E5a for the Xiaomi Mi 9.

The analysis confirms that the $C/N_{0}$ weighting scheme gives better results with respect to the elevation-dependent one for all devices, both in multi-constellation and GPS only solutions. By using the $C/N_{0}$ weighting scheme, a consistent improvement is achieved in the GPS only solution. The best improvement is showed by the Xiaomi Mi 9 (the smartphone whose observables are the noisiest) in the GPS only configuration: by switching from the elevation-dependent weighting scheme to the $C/N_{0}$ one, the three-dimensional error is more than halved, going from 22.68 m to 10.92 m. A significant improvement of 4.43 m is also shown by the Xiaomi Mi 8, while the Huawei P30 performance is improved by about 2 m. The multi-constellation approach also shows improvements for all devices; however, these improvements are smaller than those obtained in the GPS only solution. These results seem to suggest that the benefit from the $C/N_{0}$-dependent weighting scheme is better when the satellite tracking performance is worse.

The comparison between the performances obtained by the three devices also confirms what is described in the section on the analysis of the observables. The Huawei P30 shows the best performance in every configuration. In particular, the horizontal and 3D RMS errors obtained in the multi-constellation mode are equal to 3.24 m and 5.73 m, respectively of Huawei P30 with respect to the 4.14 m and 7.07 m of Xiaomi Mi 8 and the 4.90 m and 8.05 m of Xiaomi Mi 9.

Finally, regarding the evaluation of Galileo SPP positioning with E5a code observations, we found that, for Xiaomi Mi 9, the use of such signals instead of E1 ones led to a halving of the three-dimensional error: the 3D-RMS is 14.34 m using E1 measurements with respect to 7.91 m in the case of the E5a signal. The horizontal error has an even greater improvement going from 8.44 to 3.17 m. These results could be compared with that obtained by Xiaomi Mi 9 in GPS L1 only configuration with a $C/N_{0}$ weighting scheme where horizontal and three-dimensional error are of 6.82 m and 10.90 m, respectively. We report a poorer accuracy of the GPS L1 solution than the Galileo E5a one despite the fact that the smartphone tracked a slightly higher number of satellites for GPS (an average of 10.4) as compared to Galileo constellation (an average of 7.7; cf. Table 1). Such findings confirm a great benefit to standalone positioning from low-noise Galileo code observations on the E5a frequency band.

If we confer the standalone positioning performance of recent smartphones given in this work to that reported in the past papers, we discover a progress in terms of the accuracy [41]. In one of the initial studies by Sikirica et al. [42], accuracy levels of about 10 m and 20 m for horizontal and vertical components, respectively, of Huawei P10 were reported. A comprehensive assessment given in [43] revealed a diverse performance of the standalone positioning with the Samsung Galaxy series smartphones and a horizontal RMS up to 8 m for selected devices. We attribute the smartphone accuracy progress to the advancements in GNSS chips and antennas. More specifically, the results given in this paper highlighted the benefits from a multi-constellation solution, and what is even more noticeable is an application of low-noise E5a code signals that are acquired by recent dual-frequency smartphones.

## Figures and Tables

**Table 1:** Number of tracked satellites per frequency bands given as an average over the data collection session.

Smartphone	GPS		Galileo		BDS	GLONASS
	L1	L5	E1	E5a	B1	G1
Xiaomi Mi 8	9.5	3.6	4.0	4.0	7.1	5.6
Xiaomi Mi 9	10.4	4.4	8.0	7.7	6.4	8.7
Huawei P30	9.9	3.4	1.2	5.3	3.9	6.5

**Table 2:** STD of code noise normalized to undifferenced observations in the units of meters.

Smartphone	GPS		Galileo		BDS	GLONASS
	L1	L5	E1	E5a	B1	G1
Xiaomi Mi 8	1.86	0.51	1.24	0.54	1.68	4.78
Xiaomi Mi 9	3.41	0.96	2.77	0.90	2.66	5.10
Huawei P 30	0.80	0.19	0.48	0.21	0.57	2.04

**Table 3:** Three-dimensional coordinate error statistics (mean and STD) for GPS and multi-GNSS solutions with elevation and $C/N_{0}$-dependent weighting schemes.

Smartphone	GPS				Multi-GNSS			
	el. Weighting		$C/N_{0}$ Weighting		el. Weighting		$C/N_{0}$ Weighting	
	Mean	STD	Mean	STD	Mean	STD	Mean	STD
	[m]	[m]	[m]	[m]	[m]	[m]	[m]	[m]
Xiaomi Mi 8	10.61	8.25	7.77	4.57	7.50	4.63	6.13	3.52
Xiaomi Mi 9	14.85	17.15	9.62	5.15	9.87	5.71	7.21	3.58
Huawei P 30	7.72	4.73	6.25	3.38	6.72	3.73	5.07	2.67

**Table 4:** Horizontal, vertical, and 3D positioning RMS error statistics for multi-GNSS and GPS solutions with both elevation and $C/N_{0}$-dependent weighting schemes.

Smartphone	GPS						Multi-GNSS					
	el. Weighting			$C/N_{0}$ Weighting			el. Weighting			$C/N_{0}$ Weighting		
	RMS [m]			RMS [m]			RMS [m]			RMS [m]		
	Hor.	Ver.	3D	Hor.	Ver.	3D	Hor.	Ver.	3D	Hor.	Ver.	3D
Xiaomi Mi 8	8.71	10.23	13.44	5.61	7.06	9.01	4.76	7.42	8.81	4.14	5.73	7.07
Xiaomi Mi 9	11.90	19.30	22.68	6.82	8.54	10.92	16.60	9.30	11.41	4.90	6.38	8.05
Huawei P 30	5.84	6.92	9.05	4.47	5.52	7.10	4.39	6.30	7.68	3.24	4.73	5.73

**Table 5:** Coordinate statistics of Galileo E1 versus E5a-based single point positioning for Xiaomi Mi 9.

Signal	North		East		Up		Hor.	Ver.	3D
	Mean	STD	Mean	STD	Mean	STD	RMS	RMS	RMS
	[m]	[m]	[m]	[m]	[m]	[m]	[m]	[m]	[m]
E1	4.03	5.92	0.84	4.37	1.70	11.47	8.44	11.60	14.34
E5a	0.82	2.71	−0.10	1.43	5.21	5.04	3.17	7.24	7.91

## Data Availability

The data presented in this study are available on request from the corresponding author.
